# Immunotherapy for Glioblastoma: A Focus on PD-1/PD-L1 Inhibitors

**DOI:** 10.3390/cancers17233777

**Published:** 2025-11-26

**Authors:** Vasiliki Zoi, Vasiliki Galani, Chrissa Sioka, Georgios A. Alexiou, Athanassios P. Kyritsis

**Affiliations:** 1 Neurosurgical Institute, University of Ioannina, 45500 Ioannina, Greece; 2 Department of Anatomy Histology-Embryology, School of Medicine, University of Ioannina, 45110 Ioannina, Greece; 3 Nuclear Medicine, Faculty of Medicine, School of Health Sciences, University of Ioannina, 45110 Ioannina, Greece; 4 Department of Neurosurgery, University of Ioannina, 45500 Ioannina, Greece

**Keywords:** immunotherapy, glioblastoma, anti-PD-1, blood–brain barrier

## Abstract

Glioblastoma is the most common and lethal type of tumor of the central nervous system, with an average survival of 15 months after first diagnosis. Immune checkpoint inhibitors (ICIs) have been largely investigated for their ability to harness the immune system to combat tumors. However, their efficacy varies a lot depending on tumor type. In glioblastoma, PD-1/PD-L1 immunotherapy has been explored in various studies; however, the unique immunosuppressive environment in the brain and the presence of the blood–brain barrier as well as the large intratumoral heterogeneity have limited its efficacy considerably. In order to improve the clinical efficacy of ICIs, it is important to delve into the different factors affecting the response rate in GBM. Herewith, we summarize the most common causes of resistance to anti-PD-1/PD-L1 immunotherapy as well as possible ways of enhancing its efficacy, particularly through combination with other therapeutic agents in the preclinical and clinical setting. Furthermore, we provide an insight into the most promising methods for modulating the blood–brain barrier, as well as the growing role of molecular imaging and radiogenomics in this field.

## Simple Summary

Immune checkpoint inhibitors have been largely investigated in several cancers with promising results. In glioblastoma (GBM), the efficacy of immunotherapy is limited primarily due to the tumor’s immunosuppressive environment and the presence of the blood–brain barrier. This review outlines the most common causes of resistance to anti-PD-1/PD-L1 immunotherapy in GBM and mechanisms to enhance the efficacy of these agents, especially through combination with other treatment modalities and/or standard GBM therapy, as well as recently published clinical trial outcomes of GBM immunotherapy.

## 1. Introduction

Glioblastoma is the most malignant tumor of the primary central nervous system and is categorized as grade 4 glioma in the most recent WHO classification. The overall survival of GBM patients remains extremely low despite all the recent advances in the clinical environment. Nowadays, the Stupp protocol remains the standard treatment plan for GBM, consisting of surgical resection—where possible—followed by chemotherapy with the alkylating antineoplastic drug Temozolomide (TMZ) and concomitant radiotherapy. However, primarily due to the highly heterogenous nature of GBM and the implication of various signaling pathways in its pathogenesis, it still remains difficult to identify novel and effective anti-glioma therapeutic options [1].

Several studies on GBM treatment options have been performed, including the use of immune checkpoint inhibitors (ICIs). In GBM, the most important ICIs are monoclonal antibodies targeting PD-1 or PD-L1, like nivolumab and pembrolizumab. However, despite some satisfactory preclinical results, in the clinical environment, these agents fail to prolong GBM patients’ survival, thus limiting their efficiency [2]. Several biological factors are implicated in the clinical response of GBM patients to ICIs. The intratumoral heterogeneity of GBM is pronounced and, to a large extent, drives resistance to therapy. The number of heterogeneous phenotypes in GBM primarily accounts for the differentiation in the immune cell composition and the reshaping of their functions in favor of the establishment of an immunosuppressive microenvironment [3]. Therefore, a deeper understanding of GBM heterogeneity, focusing on the role of the tumor microenvironment, and its major immune cell components, including monocytes, dendritic cells, tumor-associated macrophages, and microglia is pivotal in order to design effective immunotherapeutic strategies against GBM. In this review, we explore the most common causes of resistance to anti-PD-1/PD-L1 immunotherapy in GBM, as well as possible ways of overcoming the immunosuppressive TME, primarily through the exploitation of combination therapy.

## 2. Immunotherapy for GBM

In several types of cancer, immunotherapy has become an important part of therapeutic protocols in the clinical environment. Depending on the level of immunosuppression and the ability of each tumor to overcome immune system surveillance, the success of immunotherapy may vary a lot. The brain has specific and unique immune characteristics, and under normal circumstances, it is immunologically quiescent, primarily due to the presence of the blood–brain barrier, which effectively limits the infiltration of immune cells. However, in the presence of a tumor, like GBM, pronounced immunosuppression occurs, leading to limited success of most studied immunotherapy treatments.

Immune checkpoint inhibitors (ICIs) targeting PD-1/PD-L1 have shown successful therapeutic results against several solid tumors but limited benefit in GBM. Multiple, often overlapping, mechanisms, both tumor-intrinsic and tumor-extrinsic, underlie resistance to ICIs in GBM. The most important resistance mechanisms are discussed in detail below.

### Immunosuppressive TME

One of the main causes of resistance to ICIs in GBM is the profoundly immunosuppressive tumor microenvironment (TME). Unlike other tumors, GBM grows within the central nervous system (CNS), which already possesses baseline mechanisms of “immune privilege” to limit inflammation and protect neuronal function. GBM further intensifies these mechanisms, creating a milieu that strongly suppresses cytotoxic T lymphocyte activity, even in the presence of PD-1 or PD-L1 blockade [4]. Moreover, the TME in GBM consists of several other immune cell types, with the most prominent being tumor-associated macrophages and microglia (TAMs), myeloid-derived suppressor cells (MDSCs), regulatory T cells, tumor-associated neutrophils (TANs), and dendritic cells (DC), all of which interact with each other as well as with cells secreting chemokines, cytokines, or other molecules in the TME, in favor of immunosuppression [5].

For example, TAMs constitute up to 30–50% of the GBM mass. TAMs in GBM are derived from both resident microglia and infiltrating monocytes from the circulation. Instead of supporting immune surveillance, these macrophages are “reprogrammed” by the tumor to promote growth, angiogenesis, and invasion. They secrete cytokines and growth factors that suppress cytotoxic T cell function, enhance tumor cell survival, and facilitate tissue remodeling. TAMs typically acquire an anti-inflammatory, M2-like phenotype in GBM, which is contrary to the tumor-killing, pro-inflammatory M1-like phenotype in healthy immune responses. They are responsible for secreting immunoregulatory cytokines such as interleukin-10 (IL-10), resulting in inhibition of effector T cell proliferation and promotion of the expansion of regulatory T cells (Tregs). TAMs also release vascular endothelial growth factor (VEGF) and matrix metalloproteinases, sustaining angiogenesis and tissue remodeling while perpetuating immunosuppression. Because of their abundance and significance, TAMs have become a major target for immunotherapy in GBM [6,7].

Tregs are a specialized subset of CD4+ T cells that accumulate in large numbers within the tumor and peripheral blood, where they suppress cytotoxic T lymphocytes and natural killer (NK) cells, decrease antigen-presenting cell function, and secrete inhibitory cytokines, such as IL-10 and TGF-β. This suppressive activity allows GBM cells to evade immune surveillance and contributes to tumor progression [8]. The high proportion of Tregs in GBM patients is strongly associated with poor prognosis and resistance to immune-based therapies [9]. Tregs express high levels of inhibitory receptors, such as CTLA-4, further diminishing antitumor responses. The recruitment of Tregs is facilitated by tumor-derived chemokines (e.g., CCL22, CCL28). Given their central role in maintaining GBM’s immune shield, Tregs have become an attractive target for novel immunotherapeutic approaches in GBM; however, several obstacles limit the efficacy of targeting Tregs in GBM, including the heterogeneity of their populations within GBM as well as the difficulty of selectively targeting intratumoral Tregs without affecting systemic immune tolerance [9,10].

Another important cell population in GBM is that of MDSCs, a heterogeneous population of immature myeloid cells that accumulate in both the tumor microenvironment and the circulation of patients. Circulating and tumor-infiltrating MDSCs inhibit T cell function through arginase-1 activity, nitric oxide production, and reactive oxygen species. MDSCs also promote Treg induction, reinforcing multiple immunosuppressive pathways, and they express PD-L1, resulting in additional T cell exhaustion, as well. Preclinical studies suggest that MDSC depletion or inhibition may enhance the efficacy of immunotherapy in GBM primarily by facilitating the infiltration of cytotoxic T cells into the tumor [11,12]. However, clinical translation faces several challenges. MDSCs are phenotypically diverse and overlap with other myeloid populations, making selective targeting difficult. Additionally, systemic depletion of MDSCs risks impairing normal immune homeostasis, raising important safety concerns [13].

While most of the focus in the immunosuppressive nature of GBM has been on TAMs, MDSCs, and Tregs, an increasing body of research highlights the role of tumor-associated neutrophils (TANs) as key modulators of tumor growth and response to immunotherapy as well. Specifically, TANs promote tumor progression through multiple mechanisms, including secretion of proteases that remodel the extracellular matrix, release of pro-angiogenic factors, like VEGF, and suppression of cytotoxic T lymphocytes. They also form neutrophil extracellular traps (NETs), which can create a physical barrier to immune cell infiltration and provide survival advantages to tumor cells. For these reasons, the abundance of TANs in GBM correlates with more aggressive disease and poorer prognosis [14,15].

**Figure 1:**
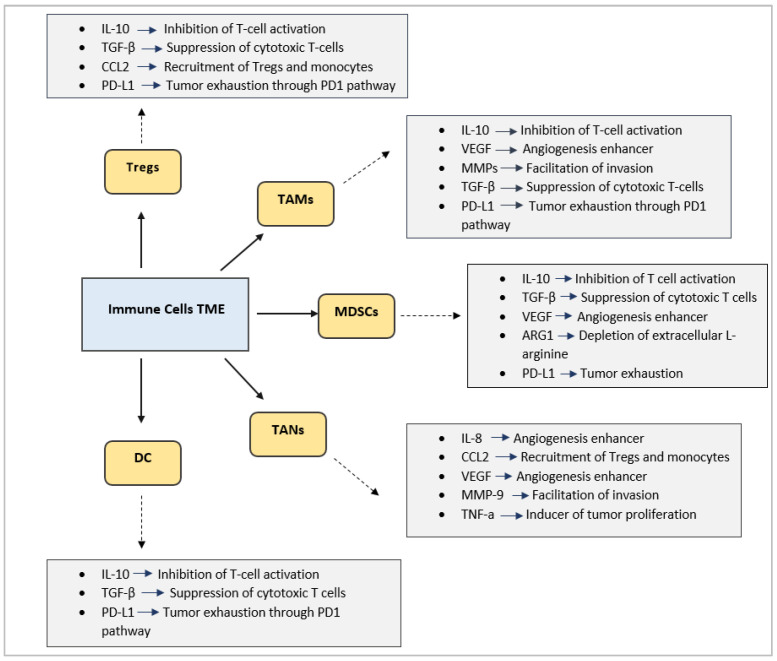
Immune cells and key secreted factors in GBM tumor microenvironment.

Lastly, it is worth mentioning the implication of DC cells in the immunosuppressive TME of GBM. Under physiological conditions, DCs present antigens to T cells, thereby initiating adaptive immune responses. In GBM, however, tumor-derived cytokines such as IL-10, TGF-β, and VEGF impair DC maturation and function. This results in poor antigen presentation, inadequate T cell activation, and as a consequence, promotion of the immunosuppressive TME. Given their central role in initiating adaptive immunity, restoring the antigen-presenting function of DCs, possibly through the development of DC vaccines or combination therapies, may help overcome GBM’s immune resistance [16]. Figure 1 summarizes the most important immune cells in the GBM tumor microenvironment and their implications in tumor progression and development of an immunosuppressive TME.

Beyond cellular components, the GBM TME is characterized by hypoxia and accumulation of metabolites such as adenosine and kynurenine. These metabolites impair T cell receptor signaling and promote T cell exhaustion. Hypoxia also induces the expression of hypoxia-inducible factor 1-α (HIF-1α), which not only promotes angiogenesis but augments PD-L1 expression on both tumor and myeloid cells as well [17].

## 3. The Role of Blood–Brain Barrier (BBB)

In GBM, the BBB becomes partially disrupted due to tumor-induced angiogenesis and inflammation. However, this disruption is heterogeneous. While some regions of the tumor may have a disrupted BBB, the infiltrating tumor margins, where cancer cells often remain after surgical resection, are usually protected by an intact barrier. This selective permeability limits the uniform delivery of immunotherapeutic agents and prevents sufficient immune surveillance across the entire tumor area [18,19].

A series of studies have focused on investigating agents that can modulate BBB permeability by targeting molecular pathways, like VEGF, claudin-5, or matrix metalloproteinases (MMPs). However, the lack of randomized clinical trials in this field remains a serious obstacle. Currently, the monoclonal antibody bevacizumab is the only immunotherapy approved for GBM targeting VEGF-A. Bevacizumab has been found to increase progression-free survival in patients with GBM; however, it fails to increase the overall survival of GBM patients in the clinical environment [20]. Different strategies have been proposed for the modulation of the BBB in order to improve immunotherapy. The most promising strategies are listed in Table 1, highlighting recent preclinical studies and the major advantages.

## 4. Combination of ICIs with Other GBM Treatments in the Preclinical Setting

Preclinical research has revealed opportunities for ICIs in GBM, especially in combination with other agents. The current consensus is that ICIs alone cannot effectively overcome the immunosuppressive properties of GBM, but rationally designed combinations hold great potential. In particular, a series of studies have evaluated the possibility of enhancing anti-PD-1 therapy when combined with chemotherapy and/or radiotherapy in GBM preclinical models. In this context, Park et al. evaluated the combinatorial effect of TMZ and anti-PD-1 in vitro and in an orthotopic murine GBM model. According to their results, combined treatment enhanced anti-GBM effects both in vitro and in vivo (complete remission of GBM in all mice compared to 44% in PD-1 monotherapy). However, combined treatment failed to induce antitumor immunological memory, resulting in tumor relapse [36]. In a similar study performed by Dai et al., an orthotopic mouse GBM model was evaluated in terms of overall survival after receiving anti-PD-1 as monotherapy versus a combination of it with TMZ. The results showed that not only was overall survival increased in the combination group but the number of CD4 and CD8 infiltrating cells was as well [37]. Interestingly, TMZ dosage has been found to be highly correlated to T cell response to ICIs in murine GBM models. As studied by Karachi et al., standard TMZ dosage combined with anti-PD-1 resulted in T cell exhaustion, whereas lower TMZ dosage enhanced the survival benefit of the anti-PD-1 therapy [38].

Recently, Aytekin et al. developed lipid carriers loaded with low TMZ doses and conjugated with anti-PD-L1. According to their results, the combination treatment considerably increased survival in an orthotopic murine GBM model. In addition, this nanostructure was able to cross the blood–brain barrier efficiently, highlighting the potential of using novel nanotechnology techniques to enhance drug delivery in the brain [39].

Apart from TMZ, radiotherapy (RT) also plays a pivotal role in the standard treatment protocol for GBM. In that regard, several scientific groups have studied the combination of RT with anti-PD-1 therapy in preclinical models of GBM. Zeng et al. investigated the combinatorial effect of anti-PD-1 treatment and stereotactic radiosurgery in an orthotopic murine model. Their results showed that combination treatment improved survival primarily due to increased tumor infiltration by T cells. Long-term survival was also observed in the group receiving combination therapy compared to monotherapies [40]. Similar results were obtained by Kim et al., who used a murine glioma model to test whether the combination of stereotactic radiosurgery, anti-PD-1, and anti-TIM-3 increased survival compared to monotherapies. The results showed that triple therapy considerably increased survival both short- and long-term [41]. The RT dosage used in preclinical models of GBM has been found to play a significant role when combined with anti-PD-1 immunotherapy. In a recent study by Cocito et al., the combination of anti-PD-1 with a single dose of 10 Gy in an orthotopic murine GBM model was more effective than the use of five consecutive doses of 2 Gy, despite the fact that the latter resembles the actual clinical protocol more [42].

Another promising preclinical combination that has been studied against GBM is anti-PD-1 therapy and anti-VEGFR2. Since angiogenesis is a major characteristic of the tumor microenvironment and has been implicated in the prevention of T cell infiltration, targeting it may facilitate immune checkpoint blockade. Indeed, as studied by Yao et al., anti-VEGFR2 therapy may enhance the therapeutic potential of anti-PD-L1 on an orthotopic murine GBM model [43]. Table 2 summarizes the most important preclinical studies of anti-PD-1 or anti-PD-L1 combinations with established anti-glioma treatments. It should be noted that although promising preclinical studies support the use of combination regimens, their mechanistic rationale varies significantly across therapeutic categories. For example, radiotherapy induces immunogenic cell death and enhances neoantigen exposure, thereby supporting T cell priming prior to PD-1 blockade. Conversely, anti-angiogenic agents normalize aberrant vasculature and reduce VEGF-mediated immunosuppression, indirectly facilitating T cell infiltration. On the other hand, standard chemotherapy can have divergent effects depending on the dosing schedule: metronomic or low-dose TMZ may preserve lymphocyte function, whereas standard high-dose TMZ often causes severe lymphodepletion that counteracts ICIs. Importantly, distinct molecular subsets of GBM may benefit differentially from combination strategies. For instance, the mesenchymal subtype is characterized by enhanced immune infiltration and may respond more favorably to ICIs, whereas the classical and proneural subtypes demonstrate weaker baseline immune activation [2,3]. Thus, future clinical trial designs should integrate biomarker-based patient stratification to match combination regimens with GBM subtypes most likely to derive benefit.

## 5. Combination Treatments in the Clinical Setting

Large randomized trials of PD-1/PD-L1 inhibitors as single agents or additions to standard therapy (primarily TMZ and/or RT) have not shown promising results in clinical trials. For example, the CheckMate 143 randomized study comparing nivolumab with bevacizumab in recurrent GBM did not show an overall survival advantage for nivolumab [44]. Furthermore, according to the results from exploratory phase I cohorts of CheckMate 143, the most common treatment-related adverse effects were fatigue and diarrhea in 30% of patients receiving nivolumab. When nivolumab was given in combination with ipilimumab, the same adverse effects were reported in 80% of the study’s patients. However, despite these toxicities, no new safety signals were identified compared to other tumors, and there was no evidence of severe neurotoxicity [45]. In another reported study, addition of nivolumab to standard chemoradiotherapy in newly diagnosed GBM patients failed to show a meaningful overall survival benefit for nivolumab in the tested populations [46]. These results helped pivot the field toward combination approaches rather than monotherapy.

Multiple early-phase clinical trials have shown that combining PD-1/PD-L1 inhibitors with other modalities (CTLA-4 blockade, temozolomide, anti-VEGF/TKIs, or myeloid-targeting agents) can be administered safely in GBM patients, with manageable immune-related adverse effects when appropriately monitored. The NCT02311920 trial established safety for ipilimumab and nivolumab when combined with standard GBM therapy and led to the larger randomized phase II/III efficacy trial (NRG BN007). According to the results of the phase 1 study (NRG-BN002), immunotherapy was well tolerated with only 16% Grade 4 events, whereas no Grade 5 events were reported in either the single-agents group or the combination group, highlighting the observation that nivolumab and ipilimumab are generally safe and tolerable [47]. Similarly, the STERIMGLI phase 1 clinical trial showed that a combination of durvalumab and hypofractionated stereotactic RT is well-tolerated in recurrent GBM patients, with only one dose limiting toxicity related to durvalumab [48]. Thus, in general anti-PD-1/PD-L1 immunotherapy is well-tolerated, and the toxicity profile in GBM is similar to what is seen in other cancers receiving ICIs. This is further supported by the results of a recent meta-analysis, reporting that PD-1/PD-L1 inhibitors in advanced cancers (beyond GBM) are better tolerated than traditional chemotherapy. In specific, immunotherapy resulted in a lower risk of common treatment-related symptoms, like diarrhea, nausea, and fatigue, compared to chemotherapy [49]. Table 3 highlights the most recent or ongoing clinical trials testing combinations of immunotherapy with other modalities in GBM patients. Although combination approaches have shown promising results in terms of patient tolerability and safety compared to monotherapies, data proving the efficacy of this approach are more limited. This can be partly attributed to a fundamental translational gap between preclinical models and human GBM. Murine GBM models typically display higher baseline immunogenicity, greater T cell infiltration, and more permeable BBB characteristics than human tumors, factors that may significantly amplify the efficacy of ICIs in the preclinical setting. In patients, however, the majority of GBMs demonstrate a profoundly “cold” immune phenotype characterized by limited cytotoxic T cell infiltration, a high percentage of immunosuppressive TAMs and MDSCs, and limited antigen presentation capacity. Therefore, in order to bridge this translational gap, thoughtful patient stratification, identification of novel biomarkers, and the development of carefully planned large-scale, multicenter clinical trials are needed.

## 6. The Growing Role of Molecular Imaging and Radiogenomics

Imaging tools play a significant role in assessing the response to immunotherapy in GBM patients. Molecular imaging and radiogenomics have emerged as transformative tools in the evaluation of GBM immunotherapy, offering novel insights into tumor biology, the immune microenvironment, and therapeutic response prediction.

Molecular imaging focuses on visualizing biological processes at a molecular and cellular level, extending beyond traditional anatomical imaging to assess tumor metabolism, immune cell dynamics, and receptor expression. Techniques such as positron emission tomography (PET) and magnetic resonance imaging (MRI) have been adapted with novel radiotracers and contrast agents targeting immune markers like PD-1/PD-L1, activated T cells, and macrophages within the tumor microenvironment. These approaches enable real-time, noninvasive monitoring of immune responses to ICIs in GBM. Recent studies on immunoPET imaging in GBM have demonstrated its powerful application in noninvasively visualizing and quantifying immune-related biomarkers, mainly focusing on CD8+ T cell infiltration and PD-L1 expression, to assess and predict immunotherapy response [57,58].

In a preclinical study by Gallegos et al., [89Zr]-CD8 ImmunoPET was used in an orthotopic murine GBM model treated with a combination of oncolytic herpes simplex virus (oHSV) and anti-PD-1 therapy. Researchers found that this dual immunotherapy significantly increased CD8+ T cell infiltration, as seen by ImmunoPET, in the tumor region compared to controls. Most importantly, longitudinal imaging revealed that responders showed a more homogeneous spatial distribution of CD8+ T cells within the tumor microenvironment, correlating with therapy success. These imaging results were validated by ex vivo analyses including autoradiography and immunohistochemistry. This study underscores the potential of ImmunoPET to serve as a noninvasive biomarker for monitoring immunotherapy efficacy and guiding treatment decisions in GBM [58]. In a similar context, Vincze et al. used an [89Zr]Zr-DFO-anti-TIGIT ImmunoPET tracer for TIGIT immune checkpoint imaging in GBM-bearing mice. TIGIT is an immune checkpoint receptor highly expressed on activated T cells. According to the results of this study, the tracer showed specific uptake in the TME but a relatively low magnitude compared to the control, highlighting both the potential and limitations of targeting TIGIT in GBM [59]. The potential of preoperative PET imaging when combined with fluorescence-guided surgery in an orthotopic GBM mouse model has also been the subject of interest for Hautiere et al., who evaluated the use of [89Zr]Zr-axiRA63-MOMIP as a theranostic approach targeting endothelin A receptors (ETA), which are highly expressed in GBM cancer stem cells [60].

Another promising imaging modality enabling precise visualization of GBM is photoacoustic imaging (PAI) combined with peptide-based strategies. In specific, short amino acid sequences, such as the transferrin-mimetic peptide T7, can selectively bind to transferrin receptors, which are overexpressed on GBM and endothelial cells, facilitating targeted drug delivery while sparing healthy tissue. Integrating these peptides with PAI, which combines optical contrast with deep tissue penetration, enables precise tumor visualization. Recent studies, including work by Zhang et al., have demonstrated that molecular probes modified with the T7 peptide can selectively accumulate in GBM cells, improving both imaging accuracy and therapeutic potential and supporting synergistic strategies such as immunotherapy [61]. Furthermore, building on this concept, all-in-one theranostic nanoprobes, such as PEG/αCD25-Cy7/TMZ, integrate targeted chemotherapy with real-time immune response monitoring. These nanoprobes selectively deliver TMZ to the tumor microenvironment while tracing regulatory T lymphocyte dynamics through combined photoacoustic–fluorescence imaging. This approach allows for noninvasive visualization of immune modulation, demonstrating increased regulatory T cell infiltration after chemotherapy and its reduction following immunotherapy. Together, peptide-targeted probes and theranostic imaging provide a powerful platform for precise drug delivery, dynamic immune monitoring, and optimized chemo-immunotherapy in GBM and thus present a novel and promising strategy that needs to be further explored in the clinical setting as well [62].

Radiogenomics is an emerging interdisciplinary field that integrates radiomic imaging features with genomic data to characterize tumor biology noninvasively. In GBM, radiogenomics preclinical studies are rapidly expanding to uncover how imaging phenotypes correlate with complex molecular landscapes. Recent preclinical research leverages advanced MRI modalities, including T1-weighted, T2-weighted, FLAIR, and contrast-enhanced scans, combined with genomic profiling of murine and patient-derived GBM models to explore oncogenic pathways and tumor heterogeneity. In a recent study by Ahanger et al., machine learning (ML) models combined with radiomic features from diverse MRI scans linked to genetic alterations in pathways such as RTK/RAS/ERK, PI3K, TP53, and NOTCH were used and evaluated. These models predicted oncogenic signaling disruptions with promising accuracy, demonstrating that phenotypic imaging traits mirror underlying molecular changes [63]. In another study by Kazerooni et al., an ML-based radiogenomic analysis of 357 IDH-wildtype glioblastomas was performed in order to identify genetic mutation patterns in multiple tumor regions. According to their results, tumors with co-occurring mutations, notably in *EGFR*, displayed distinct radiomic profiles characteristic of increased angiogenesis and proliferation [64]. Radiogenomics can also help unravel critical molecular markers correlated with therapeutic response in GBM. Methylation status of the *MGMT* promoter, a key epigenetic modification influencing chemotherapy sensitivity, has been thoroughly studied. Specifically, radiogenomic analyses have linked characteristic imaging features and changes in tumor microenvironment profiles with *MGMT* methylation, supporting its utility as a noninvasive biomarker in preclinical GBM models. These integrated data help differentiate true tumor progression from pseudoprogression, an imaging phenomenon complicating therapy monitoring, and identify patients likely to benefit from alkylating agents, like temozolomide. For example, in a study by Hegi et al., pseudoprogression was observed in 41% of patients receiving chemotherapy with unmethylated *MGMT* promoter, in contrast to 91% in patients with hypermethylation, highlighting the importance of *MGMT* methylation in therapeutic monitoring of GBM patients [65]. Another potential predictive biomarker, as studied by Zhao et al., is *PTEN*, a multi-functional tumor suppressor gene that is highly mutated in GBM patients. According to their study, non-responding GBM patients to anti-PD-1 immunotherapy had significant *PTEN* mutations compared to responders. Another finding of this study was the implication of the MAPK pathway in the efficacy of anti-PD-1 therapy. Specifically, an enrichment in mutations in major components of this pathway, including the *BRAF* gene, were observed in the responding group, highlighting the need to further investigate the implication of the MAPK pathway in the response rate to ICIs of GBM patients [66]. In another study by Hwang et al., three key immune-related genes named *TNFRSF18, TNFSF4*, and *IL12RB2* were identified as promising predictive biomarkers for responsiveness to activated NK cell therapy in recurrent glioblastoma. Specifically, all three genes were consistently upregulated in responders and showed a strong correlation with immune cell infiltration and activation patterns that were absent in the non-responding patients. Moreover, in the same study, *NOTCH1* emerged as an inverse predictive biomarker, since its expression was found to be significantly higher in non-responders. Since *NOTCH1* is known to be associated with the glioma stem cell populations, its higher expression in non-responders implies a more stem-like, therapy-resistant tumor phenotype in these patients [67]. Several other studies have also sought to unravel prognostic biomarkers that can help stratify GBM patients in response to immunotherapy. In this field, Tong et al., identified a seven-gene prognostic signature (*IGFBP2*, *CHPF*, *CTSZ*, *UPP1*, *TCF12*, *ZBTB20*, and *RBP1*) based on differentially expressed genes associated with high versus low nitrogen metabolism activity. Based on this signature, the researchers concluded that GBMs with a high “RiskScore” were enriched in immune-related pathways including TNF and NF-κB signaling and correlated with worse survival [68]. All these findings suggest that the differential expression of a series of genes may act as a predictive or prognostic tool to immunotherapy response and drug sensitivity in GBM patients.

Radiogenomic studies also highlight how mutations in genes regulating cell proliferation, apoptosis, angiogenesis, and invasion appear as distinct image-derived phenotypes. A well-studied example is that of EGFR gene amplification. When 136 GBM patients were studied by Ellingson et al., the results showed that tumors with EGFR amplification had significantly higher contrast enhancement and texture heterogeneity on MRI. Other studies have also indicated that predictive radiogenomic models using MRI data can noninvasively determine *EGFR* mutation or amplification status by analyzing imaging heterogeneity within peritumoral tissue [69,70].

## 7. Limitations and Future Directions

The immunotherapy landscape using anti-PD-1/PD-L1 agents has brought promising advances but also faces several important limitations that impact the efficacy across many tumors, including GBM. One of the most limiting factors remains resistance to therapy, occurring in a substantial proportion of patients who show no clinical response to PD-1/PD-L1 blockade. This resistance arises from multiple factors including low or heterogeneous expression of PD-L1 on tumor cells, absence of effector T cells from the tumor microenvironment, and the presence of the BBB as well as intrinsic defects in tumor immunogenicity that prevent sufficient immune activation. Moreover, among initial responders, acquired resistance frequently develops, leading to cancer progression or relapse after a few months of treatment. Mechanisms include upregulation of alternative immune checkpoints, secretion of immunosuppressive cells, such as Tregs, MDSCs, and TAMs, and the production of suppressive cytokines, like TGF-β, and interleukins, like IL-6 and IL-10. Understanding how to effectively modulate these molecular pathways to enhance the effectiveness of immunotherapy is crucial. In this direction, multi-omics approaches, including spatial transcriptomics, metabolomics, and proteomics, can help uncover different immune escape pathways and identify GBM patient populations whose molecular characteristics make them more responsive to certain immunotherapeutic approaches.

Another significant issue that needs to be further studied is the safety and tolerability profile of anti-PD-1/PD-L1 inhibitors alone or in combination with standard chemo/radiotherapy. So far, safety data from published clinical trials are encouraging, and the most commonly observed toxicities include fatigue, diarrhea, and elevated liver enzymes. Moreover, discontinuation due to toxicity is infrequent, and most adverse effects are manageable with corticosteroids or supportive care. However, a serious limitation is that very few ICIs trials in GBM patients have published robust patient-reported outcomes (quality of life, symptom burden) alongside safety. This is a broader issue in early-phase ICI trials, which often focus on clinician-assessed toxicity. Incorporating patient-reported outcomes in safety and tolerability trials is methodologically challenging, but there is growing work in oncology on how to improve this [71]. Furthermore, given the immunosuppressed state of many GBM patients, future clinical trials should stratify or select patients based on immune biomarkers (e.g., baseline lymphocyte counts, steroid use) to optimize safety. As noted in the NRG-BN002 study, many GBM patients receiving standard treatment prior to immunotherapy (RT and/or TMZ) are usually immunosuppressed, and that may mask some immune-related toxicity that would be more apparent in less immunosuppressed populations. In the same context, high baseline corticosteroid use may also influence the safety profile and should be noted in phase 1 clinical trials.

In order to improve the efficacy of anti-PD-1/PD-L1 agents, innovative strategies should be further studied and developed. To date, most immunotherapeutic strategies aim to reinvigorate exhausted T cells through immune checkpoint blockade, stimulate adaptive immunity via tumor vaccines, or introduce engineered lymphocytes directly into the tumor. Considering, however, that GBM consists of a low population of TILs, such strategies fail to provide significant therapeutic potential in the clinical setting. Consequently, future efforts should prioritize strategies that enhance TIL recruitment and effectively dismantle the dense network of immunosuppressive signals within the TME. A promising approach in this direction is combination therapy. Anti-PD-1/PD-L1 agents are increasingly being paired with other immunotherapies, targeted therapies, chemotherapy, radiotherapy, and novel modalities such as CAR-T cells. For example, combining ICIs with agents targeting other immune checkpoints (e.g., CTLA-4, LAG-3, TIM-3) aims to overcome adaptive resistance mechanisms and synergistically activate immune responses. Another matter that needs further investigation has to do with overcoming the BBB. Currently, several agents that can modulate BBB permeability by targeting molecular pathways are under investigation, as well as alternative physical or chemical methods to bypass this barrier. The development of novel drug-delivery strategies such as nanocarriers, focused-ultrasound-mediated BBB opening, and ligand-targeted transcytosis could improve intratumoral delivery of ICIs. Another strategy that is worth mentioning is the use of tumor-treating fields (TTFs), which employ low-intensity, intermediate-frequency alternating electric fields to inhibit tumor growth. These fields are believed to interfere with mitotic spindle formation, resulting in disrupted cell division and subsequent mitotic arrest. Recently, the early results from a multicenter randomized trial (NCT00916409) combining TTFs with maintenance TMZ demonstrate a clear benefit: median progression-free survival increased to 7.1 months with TTFs plus TMZ versus 4 months with TMZ alone, and median overall survival improved to 20.5 months compared to 15.6 months. Importantly, this localized therapy did not lead to greater systemic toxicity or increased seizure frequency relative to TMZ monotherapy, although patients did experience a higher rate of scalp irritation, as well as symptoms such as anxiety, confusion, insomnia, and headache. Moreover, the combined use of TTFs and anti-PD-1 immunotherapy appears to generate a powerful in situ vaccination effect in GBM by coupling TTFs-induced tumor cell inflammasome activation with systemic immune reinforcement from pembrolizumab [72,73].

Equally important is the identification of robust biomarkers that can predict which subpopulations of GBM patients are most likely to benefit from specific immunotherapeutic strategies. Considering the profound intratumoral heterogeneity of GBM, identification of novel biomarkers and subtype-tailored treatment strategies may ultimately help maximize clinical benefit. In this context, radiogenomics has the potential to uncover how imaging phenotypes correlate with complex molecular landscapes in order to help identify key biomarkers that influence responsiveness to immunotherapy and thus serve as a personalized decision-making tool, selecting patients most likely to benefit from immunotherapy, monitoring early treatment response, and detecting resistance mechanisms long before they appear on conventional imaging. Therefore, harnessing advanced technologies and multidisciplinary modalities promises to maximize the clinical benefits of immunotherapy in GBM, ultimately improving survival and quality of life for a broader population of cancer patients.

## 8. Conclusions

Immunotherapy has shown promising results in the treatment of different solid tumors. However, in GBM, surpassing the immunosuppressive nature of this tumor and its microenvironment remains a challenge. PD-1/PD-L1 inhibitors as a monotherapy do not alter the course of GBM for most patients. However, their safety and tolerability in GBM patients have been well-established so far. Therefore, it is important to receive data from large-scale clinical trials focusing on ICIs in combination with other anti-glioma modalities and/or radiochemotherapy. That way, novel strategies could be further developed to help overcome immunosuppression and resistance to therapy. In conclusion, ICIs for GBM face multiple challenges, and thus, more in-depth studies and clinical trials are needed.

## Figures and Tables

**Table 1:** Methods to modulate BBB in GBM, their major advantages, and recent preclinical studies.

Method	Major Advantages	Preclinical Studies	Ref.
Focused Ultrasound (FUS) + Circulating Microbubbles	-Consistent, reproducible, transient BBB opening -Noninvasive -No serious damage to the brain tissue -Can enhance delivery of ICIs -Precise targeting via MRI guidance	-Increase in CD4^+^ and CD8^+^ T cell infiltration in C6 glioma rats when combined with anti-PD-1.	[21]
		-Increased etoposide delivery in an orthotopic pontine glioma model.	[22]
		-Increase in the tumor-infiltrating lymphocyte population after combination of FUS-induced BBB opening and IL-12 in C6 glioma rats.	[23]
		-Enhanced radiotherapy effects, including increase in apoptosis of tumor cells in an F98 rat glioma model.	[24]
		-Increased survival and immune memory in orthotopic GL261 and CT-2A model after combination of FUS/MB with doxorubicin and anti-PD-1 therapy.	[25]
Receptor-Mediated Transcytosis (RMT)	-Tumor selectivity -Delivery of various drug types, like peptides, antibodies, and drug-loaded nanoparticles -Fewer systemic side effects	-Increased BBB penetration and tumor uptake in orthotopic glioma models when Angiopep-2 (LRP1)–paclitaxel conjugate was evaluated.	[26]
		-Increased antitumor activity in both subcutaneous and orthotopic GBM models of a TfR-targeted aptamer–drug conjugate (ApDC).	[27]
		-Reduction in tumor volume and preferential invasion in the tumor microenvironment of an F98 glioma rat model when LDLR ligand-functionalized gold nanoparticles were used.	[28]
		-Increased BBB penetration and tumor uptake in an orthotopic glioma model when an αvβ integrin and NRP-1-mediated transport was achieved using iRGD modified polymeric micelles.	[29]
Transporter-Mediated Transcytosis (TMT)	-Tumor selectivity -Delivery of various drug types, like peptides, antibodies, and drug-loaded nanoparticles -Fewer systemic side effects	-Ιnduction of tumor cell apoptosis and reduced tumor burden in an orthotopic GBM model when GLUT1-mediated BBB permeabilization of magnetite NPs with arginine modification was achieved.	[30]
		-Increased tumor accumulation of LAT1-targeting nanoparticles co-loaded with TMZ and sorafenib in an orthotopic GBM model.	[31]
		-Improved BBB-crossing capability in an orthotopic glioma tumor model of a smart polymer that crosses the BBB via choline transporters.	[32]
Efflux Transporter Inhibitors	-Broad applicability -Sensitization of tumor-initiating cells/GBM stem-like cells	-Increased tumor uptake of ispinesib (P-gp/Bcrp substrate) after co-administration with the dual P-gp/BCRP inhibitor elacridar in orthotopic GBM models.	[33]
		-Increased brain TMZ levels and higher antitumor effects after genetic knockout of Abcb1a/b and Abcg2 in intracranial mouse models.	[34]
Cell-penetrating peptide (CPP)–Drug Conjugates	-Enhanced solubility and bioavailability -Versatile delivery -Targeting and specificity	-Increased platinum levels in a murine GBM xenograft model and increased survival after administration of a Pt complex conjugated to a brain-penetrant macrocyclic peptide.	[35]

**Table 2:** Preclinical evaluation of different combinations of immunotherapy and other treatments against GBM in orthotopic murine GBM models.

Combination	Key Findings	Ref.
anti-PD-1 + TMZ	• Complete remission in all mice with combination (vs. 44% with anti-PD-1 alone) • No induction of immunological memory → tumor relapse	[36]
	• Increased overall survival; higher CD4+ and CD8+ T cell infiltration • Combination enhanced immune infiltration	[37]
	• Standard TMZ dose → T cell exhaustion • Low-dose TMZ → improved survival with anti-PD-1	[38]
nanocarriers (anti-PD-L1 + TMZ)	• Increased survival; efficient blood–brain barrier penetration • Nanotechnology-based delivery improved outcomes	[39]
anti-PD-1 + RT	• Improved survival; enhanced T cell infiltration • Long-term survivors only in combination group • RT synergizes with ICI	[40]
	• 10 Gy × 1 + anti-PD-1 more effective than 5 × 2 Gy • Single high dose outperforms fractionated regimen	[41]
anti-PD-1 + RT + anti-TIM-3	• Significant short- and long-term survival benefit • Triple therapy superior to monotherapies	[42]
anti-PD-L1 + anti-VEGFR2	• Increased survival via improved T cell infiltration • Targeting angiogenesis enhances ICI efficacy	[43]

**Table 3:** Completed or ongoing clinical trials testing combinations of immunotherapy with other modalities against GBM.

Phase/ID	Population	Tested Combinations	Status	Key Results	Ref.
III NCT02667587	Newly diagnosed GBM (MGMT methylated promoter) patients	Nivolumab + RT ± TMZ	Completed	➢ No improvement in OS or PFS of the combination	[50]
I/II NCT02866747	Recurrent and newly diagnosed GBM patients	Durvalumab + hypofractionated RT	Completed	➢ Combination was well tolerated	[49]
I/II NCT03174197	Newly diagnosed GBM patients	Atezolizumab (PD-L1 + temozolomide + radiation)	Active—not recruiting	➢ Benefit in OS after combination treatment, association with immune, mutation, and gut microbiome features; pending results	[51]
I/II NCT05039281	Recurrent GBM patients	Atezolizumab + cabozantinib (TKI)	Recruiting/early phase	➢ Objectives: safety profile of combination, OS, PFS; first results: well tolerable combination, no safety signals	[52]
II (early) NCT04729959	Recurrent GBM patients	Atezolizumab + tocilizumab (IL-6R inhibitor) + SRT	Active—not recruiting	➢ Combining PD-L1 blockade with myeloid/IL-6 pathway blockade + RT to overcome immunosuppression; no outcome data yet.	[53]
I NCT03961971	Recurrent GBM patients	Spartalizumab + MBG453 (anti-TIM-3) + SRT	Active—not recruiting	➢ Combining PD-L1 blockade with monoclonal antibodies, such as MBG453, to test tolerability and safety; no outcome data yet.	[54]
I NCT04656535	Recurrent GBM patients	Zimberelimab + domvanalimab (anti-TIGIT)	Active—not recruiting	➢ Combining PD-L1 blockade with anti-TIGIT to test tolerability and safety; no outcome data yet.	[55]
I NCT02658981	Recurrent GBM patients	Nivolumab + BMS-986016 (anti-LAG-3)	Completed	➢ Combination cohorts showed manageable safety; full efficacy conclusions pending larger randomized studies	[56]

Abbreviations: IL-6R, interleukin-6 receptor; LAG-3, lymphocyte-activation gene 3; MGMT, O6-methylguanine-DNA methyltransferase; OS, overall survival; PFS, progression-free survival; RT, radiotherapy; SRT, stereotactic radiotherapy; TIM-3, T cell immunoglobulin and mucin domain 3; TKI, tyrosine kinase inhibitors, TMZ, temozolomide.

## Abbreviations

The following abbreviations are used in this manuscript:

BBB	Blood–Brain Barrier
CNS	Central Nervous System
DC	Dendritic Cells
GBM	Glioblastoma
HIF-1a	Hypoxia-Inducible Factor 1a
ICIs	Immune Checkpoint Inhibitors
IL-6R	Interleukin-6 Receptor
IL-10	Interleukin-10
LAG-3	Lymphocyte Activation Gene-3
MDSCs	Myeloid-Derived Suppressor Cells
MGMT	Methylguanine-DNA Methyltransferase
MMPs	Matrix Metalloproteinases
MRI	Magnetic Resonance Imaging
NETs	Neutrophil Extracellular Traps
NK	Natural Killer
OS	Overall Survival
PET	Positron Emission Tomography
PFS	Progression-Free Survival
RT	Radiotherapy
SRT	Stereotactic Radiotherapy
TAMs	Tumor-Associated Macrophages
TANs	Tumor-Associated Neutrophils
TIM-3	T cell Immunoglobulin and Mucin Domain 3
TKI	Tyrosine Kinase Inhibitor
TME	Tumor Microenvironment
TMZ	Temozolomide
TTFs	Tumor-Treating Fields
VEGF	Vascular Endothelial Growth Factor
